# The faces behind vaccination: unpacking the attitudes, knowledge, and practices of staff of Cameroon’s Expanded program on Immunization

**DOI:** 10.1186/s12960-023-00869-7

**Published:** 2023-11-13

**Authors:** Yauba Saidu, Jessica Gu, Budzi Michael Ngenge, Sangwe Clovis Nchinjoh, Amani Adidja, Nadege Edwidge Nnang, Nkwain Jude Muteh, Vouking Marius Zambou, Clarence Mvalo Mbanga, Valirie Ndip Agbor, Diaby Ousmane, Andreas Ateke Njoh, Junie Flegere, Demba Diack, Owens Wiwa, Emanuele Montomoli, Sue Ann Costa Clemens, Ralf Clemens

**Affiliations:** 1 Clinton Health Access Initiative Inc., PO Box 2664, Yaounde, Cameroon; 2 Institute for Global Health, University of Siena, 53100 Siena, Italy; 3 Global Vaccine Delivery, Clinton Health Access Initiative Inc, Boston, MA 02127 United States of America; 4 Faculty of Medicine and Biomedical Sciences, University of Yaoundé 1, Yaoundé, Cameroon; 5 Gavi, The Vaccine Alliance, Geneva, Switzerland; 6 UNICEF, Yaoundé, Cameroun; 7 Clinical Trial Service Unit and Epidemiological Studies Unit (CTSU), Nuffield Department of Population Health, University of Oxford, Oxford, United Kingdom; 8 Department of Projects, Ministry of Public Health, Yaounde, Cameroon; 9 Expanded Program on Immunization, Cameroon Ministry of Public Health, PO Box 2084, Yaoundé, Cameroon; 10 School of Global Health and Bioethics, Euclid University, PO Box 157, Bangui, Central African Republic; 11 Clinton Health Access Iniative Inc., Abuja, Nigeria; 12 Department Molecular Medicine, University of Siena, Via Aldo Moro 3, 53100 Siena, Italy; 13 VisMederi Srl, Via Ferrini 53, 53035 Siena, Italy; 14 Department of Pediatrics, University of Oxford, Oxford, United Kingdom

**Keywords:** Expanded Program of Immunization, Knowledge, Attitudes, Practices, Vaccination staff, Cameroon

## Abstract

**Background:**

Immunization is regarded as one of the most cost-effective public health interventions in global health. However, its cost-effectiveness depends greatly on the knowledge and skills of vaccinators. With the growing complexity of immunization programs, the need for a well-trained vaccination workforce cannot be overemphasized. In this study, we assessed the knowledge, attitudes, and practices among vaccination staff in Cameroon.

**Methods:**

Through a descriptive cross-sectional design, we used structured questionnaires and observation guides to collect data from vaccination staff in health facilities that were selected by a multistage sampling method. Data were analyzed using STATA 13 software.

**Results:**

Overall, we collected data from Expanded Program on Immunization focal staff in 265 health facilities across 68 health districts. Over half (53%) of the surveyed facilities were found in rural areas. Nearly two-thirds of health facilities had immunization focal staff with knowledge gaps for each of the four basic immunization indicators assessed. In other words, only 37% of staff knew how to estimate coverages, 36% knew how to inteprete the EPI monitoring curve, 35% knew how to prepare vaccine orders, and 37% knew how to estimate vaccine wastage. In terms of practices, staff waited for more than ten children to be present before opening a 20-dose vaccine vial in 63% of health facilities, and more than five children to be present before opening a 10-dose vaccine vial in 80% of surveyed facilities. Provision of vaccine-specific information (informing caregiver about vaccine received, explanation of benefits and potential side effects) during immunization sessions was suboptimal for the most part.

**Conclusion:**

This study suggests marked deficits in immunization knowledge among vaccination staff and exposes common attitudes and practices that could contribute to missed opportunities for vaccination and hinder vaccination coverage and equity in Cameroon. Our findings highlight the urgent need to invest in comprehensive capacity building of vaccination staff in Cameroon, especially now that the immunization program is becoming increasingly complex.

## Introduction

Immunization is regarded as one of the most cost-effective public health interventions in modern public health history. This intervention alone averts between 3.5 to 5 million deaths annually [1] and has substantially contributed to the observed reduction in global child mortality, from 12.5 million under-five deaths in 1990 to 5.3 million deaths in 2018 [2]. Over the past decades, great strides have been made globally in expanding the reach of immunization programs; however, the coverage of the third dose of the diphtheria–tetanus–pertussis, containing vaccine (DTP-3) has not gone above 86% since 2018 [3]. Similarly, despite strides in improving the performance of routine vaccinations, coverage in the WHO African region has stagnated for about a decade, with significant inter- and intra-country disparities [4–6].

The expanded program of immunization (EPI) in Cameroon, which was launched in 1976, has remarkably contributed to increased vaccination coverage over the past four decades [7]. In 2010, Cameroon was one of the three Central African countries with an immunization coverage rate of over 80% [8]. Despite this remarkable progress, national immunization coverage still falls below set targets. In Cameroon's 2015 to 2019 comprehensive multi-year plan (cMYP), the EPI envisaged raising the DTP-3 coverage from 89% in 2013 to 92% in 2019. Unfortunately, the EPI did not only fail to attain this goal, the program registered a 22 percent point decrease, with DTP-3 coverage plummeting from 89% in 2013 to 67% in 2019 [6, 9]. This drop in immunization coverage has left many children without life-saving vaccines. Indeed, according to the most recent Demographic and Health Survey (DHS) in Cameroon, only 52% of children aged 12–23 months have received all essential vaccinations (one dose of Bacille Calmette-Guerin, BCG and measles vaccines and three doses of DTP and poliomyelitis vaccines) [10]. In addition, in 2019, Cameroon was among the top 10 countries contributing to 86% of the world's 7.3 million estimated zero-dose children [11].

Immunization coverage has been shown to be driven by several factors [12, 13]. Indeed, a well-functioning routine immunization system relies on interactions of several components, including robust cold chain and logistics management systems, sustainable financing, strong managerial and technical leadership, and quality service delivery [14]. In addition, a well-functioning vaccination system with quality vaccination services anchors on effectiveness as a core guiding principle, defined by the World Health Organization as providing evidence-based vaccination services based on scientific rigor to achieve the best possible outcomes [15]. While the community needs to collaborate with healthcare providers to improve coverage, the quality of vaccination services provided by health personnel is imperative for the success of vaccination programs [16]. This success has been shown to significantly depend on the knowledge, attitudes, and skills of vaccination and managerial staff at healthcare facilities [17]. Indeed, the need for a well-trained and competent health workforce for vaccination cannot be overemphasized, particularly in recent years where immunization programs are bent on "leaving no one behind" and expanding the benefits of vaccination to every individual, irrespective of who he/she is and where he/she lives [18]. Meeting this noble goal will require significant improvements in providers' knowledge, attitudes, and practices, as these could positively or negatively influence parental decisions to seek vaccination services or return for subsequent vaccinations. For example, Musa et al. reported increased immunization service utilization in settings where health workers displayed positive attitudes and practices [19]. However, such evidence is limited in sub-Saharan Africa. As a result, evaluating the knowledge, attitudes, and practices of vaccination staff may serve as a standpoint for improving the quality of immunization service delivery, which in turn can improve immunization coverage and equity in many settings.

In Cameroon, several studies have examined specific drivers of declining routine immunization (RI) performance [20–24]. However, none of the work focused on assessing immunization knowledge among vaccination staff and their attitudes and practices during vaccination sessions. Thus, this study aimed to generate preliminary data on this neglected area of immunization.

## Materials and methods

### Study design

This descriptive cross-sectional study was based on data from a national baseline assessment that was implemented by the EPI in collaboration with the Clinton Health Access Initiative (CHAI). The study aimed at identifying and characterizing potential factors contributing to declining immunization coverage in Cameroon.

### Study setting

The study was conducted in Cameroon, a country that is located in the Gulf of Guinea. The country has a population of approximately 28 million inhabitants and a total surface area of 475,440 km^2^ [25]. The country is divided into ten administrative regions: Adamawa (AD), Center (CE), East (ES), Far-North (EN), Littoral (LT), North (NO), North West (NW), West (OU), South (SU) and South West (SW) regions [26].

Cameroon's health sector is organized into three main levels (central, intermediate, and peripheral), each having specific competencies, administrative, health, and dialogue structures. The health structure lies under the leadership of the Minister of Public Health. The central level is led by various directorates under the leadership of the Minister of Public Health and focuses mainly on developing policies, strategies, and coordination. The intermediate level is led by the 10 Regional Delegates and provides technical support to the 189 health districts nationwide. District Medical Officers manage the health districts at the third level, the operation or implementation level for primary health care in Cameroon. Preventive services, including, immunization activities, are incorporated into all health system levels [27].

### Sampling

A multistage sampling technique was used to select health facilities. Before sampling, the total number of districts was allocated proportionately to the total number of districts per region in the ten regions. Then, the number of urban and rural districts was assigned within each region based on the region-specific breakdown and health facilities were allocated across regions in proportion to the national distribution.

The districts were then randomly selected within the specified region's urban or rural strata in the first stage. Health facilities were randomly selected within the identified rural/urban districts in the second stage. This selection was made while ensuring that the same number of facilities was selected within each district.

### Study procedures

### Administrative approval

Administrative approval was obtained from the ministry of public health before data collection. Additionally, written approvals were also obtained from all regional delegations of public health, who in turn issued administrative letters to district heads requesting full support for the data collection process.

#### Training

Training was carried out in all regions to provide regional supervisors and data collectors with the necessary knowledge and skills to undertake the baseline assessment. The training consisted of theoretical presentations and practical sessions on data collection, entry, and transmission processes. During practical sessions, assessors were split into groups and accompanied by the assessment management team to health facilities, where they were closely observed as they completed questionnaires and observation guides.

#### Data collection

Data collection was conducted by trained assessors selected from regional and district staff. The assessment management team and regional EPI teams developed data collection plans for target districts and health facilities. To prevent unproductive visits during data collection (e.g., visiting a health facility when there was no vaccination session), assessors contacted the health facilities via phone to remind them of planned visits. Upon arrival in the facilities, the purpose of the assessment was explained to the facility head or their representative. Then the assessor first obtained informed written consent before proceeding to interview the health provider in charge of immunization service delivery in each facility and observed an ongoing fixed post-vaccination session.

#### Study tools

The tools used for this study were:

- A health facility questionnaire designed to assess the knowledge, attitudes and practices of immunization staff and their knowledge of key immunization indicators.
- Vaccination Services Observation Guide, which included several prompts to assess the attitudes and practices of vaccinators during immunization sessions.

These study tools were developed in English and French and pre-tested in four facilities in Yaounde prior to study initiation.

#### Data management and analysis

Before data entry, a comprehensive database was built, pre-tested, and validated by an expert data manager. Each assessor entered data from the filled questionnaires and observation forms into the database and transmitted the files to a secure server within three days of data collection. Data were exported and cleaned in Microsoft Excel 2016 and analyzed with STATA 13 software (StataCorp. 2013. *Stata Statistical Software*: Release 13. College Station, TX: StataCorp LP). Frequencies and proportions were used to summarize variables of interest, and the unit of analysis was the health facility. Districts and facilities were sampled proportional to national distributions, and no post-stratification weights were applied.

#### Operational definition of variables

Immunization staff knowledge: This was defined as the knowledge of the health provider in charge of immunization service delivery (EPI focal point) on four key immunization indicators (vaccination coverage estimation, Interpretation of the EPI curve, preparation of a vaccine order, and estimation of vaccine wastage). Knowledge on each of these indicators was assessed separately, as either correct or incorrect based on EPI recommendations.

Immunization staff attitudes and practices: This was based on assessors' observation during fixed post-immunization sessions and the responses from the EPI focal point's interview.

Immunization staff: Included all health staff working in the immunization unit. Only the head of the immunization unit (or their representative) was interviewed for knowledge assessment.

## Results

### General characteristics of health facilities

A total of 265 health facilities in 68 health districts were assessed nationwide during the study period. Over half (53%) of the facilities were in rural areas. Of all the facilities surveyed, the Center (21%), Littoral (13%), and North West (13%) regions were most represented, as shown in Table 1.

**Table 1:** General characteristics of surveyed health facilities

Regions	AD	CE	ES	EN	LT	NO	NW	SU	SW	OU	NAT
Distribution of health facilities											
Rural (N)	8	22	14	10	12	8	26	19	8	13	140
Urban (N)	3	34	7	6	22	8	9	13	6	17	125
Total (N)	11	56	21	16	34	16	35	32	14	30	265
% of HCW working in immunization per health facility											
Less than 2	11	9	23	4	16	0	24	31	33	7	16
2–5	67	86	65	91	77	83	61	54	55	90	74
More than 5	22	5	12	5	7	17	15	15	12	3	10
% of trained HCW per health facility											
None	78	66	100	68	59	84	49	93	76	61	67
1–2	22	30	0	23	26	17	32	8	12	23	22
3–5	0	2	0	9	13	0	15	0	6	13	7
More than 5	0	2	0	0	13	0	5	0	6	3	4

*NAT* National, AD Adamawa, *CE* Center, *ES* East, *EN* Extreme North, *LT* Littoral, *NO* North, *NW* North West, *SU* South, *SW* South West, *OU* West, *HCW* health care worker, *HF* health facility, *VC* vaccination coverage

The majority (84%) of the health facilities had two or more staff assigned to the immunization service unit, though with notable regional disparities. Notably, in the South region, 31% of health facilities had only one health provider assigned to vaccination services. Over two-thirds (93%) of the facilities had no trained immunization staff, with facilities in the South (85%) and East (100%) having very high proportions of untrained staff.

### Vaccine-provider knowledge on key immunization indicators

**Fig. 1:**
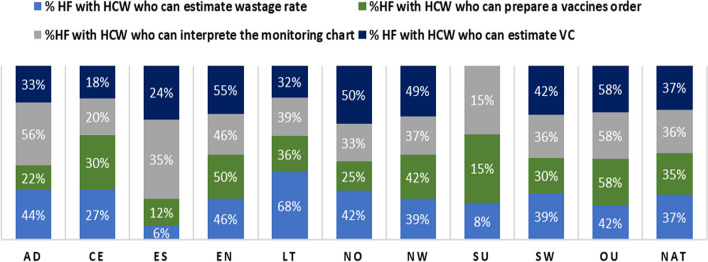
Regional distribution of vaccine-provider knowledge on key immunization indicators. *NAT* National, *AD* Adamawa, *CE* Center, *ES* East, *EN* Extreme North, *LT* Littoral, *NO* North, *NW* North West, *SU* South, *SW* South West, *OU* West, *HCW* health care worker, *HF* health facility, *VC* vaccination coverage

Figure 1 provides level of awareness of selected immunization indicators. Overall, only 37% of health facilities had immunization staff who knew how to estimate vaccination coverage. In most regions, less than a third of health facilities had staff knowledgeable on vaccination coverage estimation. It is worth noting that in the South region, no surveyed facility had staff who had knowledge on estimating vaccination coverage. Figure 1 also shows that at national level, only 36% of health facilities had staff who could interpret the EPI monitoring curve, with the West (58%) and Adamawa (56%) regions having the highest proportion of such staff. It was also noted that only 35% and 37% of health facilities had staff knowledgeable on preparing a vaccination order and estimating vaccine wastage, respectively (Fig. 1).

### Healthcare workers attitudes and practices

Table 2 provides a battery of attitudes and practices of immunization staff that were observed by study staff during immunization sessions in the 265 surveyed facilities. In terms of vaccine handling and utilization, HCWs in 46% of facilities waited for 10 to 15 children to be present for vaccination sessions before a 20-dose BCG vial was opened, while in 17% of facilities, staff waited for more than 15 children to be present. Similarly, in 68% of health facilities, HCWs waited for five to nine children to be present before opening a 10-dose pentavalent or measles vaccine vial, while 21% of facilities waited for at least nine children before opening a 10-dose vial. In all facilities in the Far North and North regions, HCW did not open a vial if less than 5 children were present for vaccination.

**Table 2:** Attitudes and practices of immunization staff during service delivery (%)

Regions	AD	CE	ES	EN	LT	NO	NW	SU	SW	WE	NAT
HCW opens a 20-dose vial when the following number of children are present:											
< 10 children	33	21	6	23	35	25	36	23	39	28	28
10–15 children	67	55	46	36	58	33	37	62	21	55	46
> 15 children	0	23	48	41	6	42	27	15	40	17	26
HCW opens a 10-dose vial when the following number of children are present:											
< 5 children	22	9	6	0	9	0	24	16	6	13	11
5–9 children	67	62	46	72	87	83	54	85	73	81	68
> 9 children	11	29	48	28	3	16	22	0	21	6	21
HCW checks the child's age before vaccination:											
Yes	89	95	100	83	88	88	100	92	100	97	94
No	11	6	0	17	12	12	0	8	0	3	6
HCW places vaccines on icepacks during the session:											
Yes	11	55	53	54	47	65	35	31	23	35	44
No	89	45	47	46	53	35	65	69	77	65	56
HCW writes the date that the multi-dose vial was opened:											
Yes	11	30	13	8	12	12	32	8	14	16	18
No	89	70	87	92	88	88	68	92	86	84	82
HCW checks the vaccines that the child received previously:											
Yes	89	88	93	83	91	71	100	92	95	84	89
No	11	12	7	17	9	29	0	8	5	16	11
HCW informs the parent/caregiver what vaccine the child is receiving:											
Yes	67	52	40	33	50	24	62	31	59	68	51
No	33	48	60	67	50	76	38	69	41	32	49
HCW informs the parent/caregiver of the benefits the vaccine:											
Yes	56	53	13	29	38	35	62	46	59	68	49
No	44	47	87	71	62	65	38	54	41	32	51
HCW informs the parent/caregiver of the normal side effects associated with the vaccine:											
Yes	22	45	13	33	44	12	21	15	27	39	32
No	78	55	87	67	56	88	79	85	73	61	68
HCW informs the parent/caregiver of other side effects associated with the vaccine?											
Yes	22	36	20	25	38	6	24	23	32	48	31
No	78	64	80	75	62	94	76	77	68	52	69
HCW encourages parents/caregivers to return for follow-up vaccination:											
Yes	78	89	93	79	91	76	82	92	86	87	86
No	22	11	7	21	11	24	18	0	14	13	14
HCW is polite to caregivers (e.g., smiling and not yelling)?											
Yes	89	95	93	79	91	88	94	92	95	97	92
No	11	5	7	21	9	12	6	8	5	3	8

*HCW* Healthcare worker, *NAT* National, *AD* Adamawa, *CE* Center, *ES* East, *EN* Extreme North, *LT* Littoral, *NO* North, *NW* North West, *SU* South, *SW* South West, *OU* West, *HCW* health care worker, *HF* health facility, *VC* vaccination coverage

Practices regarding handling of multi-dose vials were concerning. Overall, HCW in 18% of the surveyed facilities noted the date that a WHO-MDVP was opened—an observation that was consistent across all 10 regions (Table 2). Another harmful practice was placing vials on icepacks. Indeed, in 44% of surveyed facilities, HCWs placed vials on ice packs during immunization sessions, and this practice was particularly prominent in the North (65%), Center (55%), Far North (54%) and East (53%) regions.

Table 2 also illustrates some key parameters that were checked by HCW before vaccine administration. As illustrated, HCW in 94% of surveyed facilities verified the ages of children before administering a vaccine—a finding that was consistent across the 10 regions. Similarly, HCWs in nearly 90% of facilities verified the vaccines that the child had previously received before administering the next one. Despite these positive practices, HCW in 49% of health facilities, did not inform caregivers about the vaccine their child was receiving. Similarly, HCW in 51% of surveyed health facilities did not educate caregivers about the benefits of vaccination. Similarly, HCWs did not inform the caregivers about normal and potential side effects of vaccination in 68% and 69% of surveyed facilities, respectively. Last, but not least, HCWs in 14% of facilities did not request caregivers to return for follow-up vaccinations.

## Discussion

This study, which was nested in a national baseline assessment of Cameroon's immunization system, aimed at examining the immunization knowledge of vaccination staff in Cameroon as well as their attitudes and practices during vaccination sessions. We found that the knowledge of immunization staff on vaccination in practice was limited, with remarkable regional disparities. In assessing staff attitudes and practices during vaccination sessions, we noted significant gaps in health worker-to-caregiver communication and the utilization and handling of multi-dose vaccines. To the best of our knowledge, this is the first study evaluating immunization staff's knowledge, attitudes, and practices on a national scale in Cameroon.

We found that for the four basic immunization indicators assessed (vaccination coverage estimation, EPI monitoring chart interpretation, vaccine order preparation, and vaccine wastage estimation), nearly two-thirds of health facilities had focal immunization staff with suboptimal knowledge. This observation corroborates the findings of a study carried out in one district in Cameroon, which noted limited vaccination knowledge of health personnel using a different set of immunization indicators [16]. Another study in Nigeria reported that only 55% of vaccinators in 54 surveyed facilities were familiar with WHO-MDVP for minimizing vaccine wastage [28]. A poor knowledge base could limit immunization staff's ability to plan and deliver quality vaccination services, thus hindering improvement in vaccination coverage. One of the factors that could be at the root of this knowledge deficit in our study is the limited capacity building of staff working in vaccination services, as up to 67% of the surveyed facilities had no staff trained on immunization. This issue highlights the need for regular capacity building of the vaccination workforce as immunization programs become more complex with the increasing number of vaccines and recommendations [14]. Training programs have been shown, in different settings, to increase the knowledge of primary care health workers involved in vaccination and improve vaccination coverage [29, 30].

As pertains to the utilization of multi-dose vaccines, vaccine providers waited for more than 10 children to be present before opening 20-dose vaccine vials in over three-quarters (75%) of health facilities, and more than 5 children to be present before opening 10-dose vaccine vials in a great majority (91%) of surveyed facilities. This practice is comparable to findings from a study in Nigeria which found that, on average, vaccinators waited for a minimum of six children to be present before opening a 10-dose measles-containing vaccine (MCV) [28]. Similarly, in a multi-country qualitative study, some healthcare workers in Senegal and Zambia reported sending unvaccinated children back home because not enough children were present to necessitate the opening of a new 10-dose vial [31]. These attitudes and practices with multi-dose vaccines could lead to increased caregiver waiting time, influencing their decisions for future vaccinations, and contribute to missed opportunities for vaccination (MOV) either directly or indirectly, which in turn may impact vaccination coverage [32]. Mindful that the objective usually driving these practices is to minimize vaccine wastage and prevent stockouts, these findings underscore the need to put policies and strategies in place to ensure reducing MOV is prioritized over wastage concerns [30]. As a result, many countries are considering switching to products with smaller dose vials; however, such a switch could overwhelm the cold chain and supply chain capacity of the vaccination system and significantly increase the cost of vaccination per child [33]. This finding was even more surprising for vaccine products that meet the four critical criteria for the WHO Multi-Dose Vial Policy that allows for the storage of open vials for up to 28 days [34]. However, this could be accounted for by high staff attrition, particularly in private facilities in urban areas—further highlighting the importance of putting in place a system for continuous learning, including onboarding, e-learning, coaching, and supportive supervision.

Another remarkable finding was that vaccine providers placed vials on ice packs during vaccination sessions in nearly half (44%) of health facilities. This practice could compromise the potency of freeze-sensitive vaccines. Exposure of vaccines to negative temperature is pervasive in Cameroon, not only limited to immunization sessions but across the entire supply chain, from central vaccine stores to outreaches [23]. Again, this is a capacity issue that should be corrected with training to improve their vaccine handling knowledge and practices.

We also found that providing vaccine-specific information (informing caregiver about vaccine received, explanation of benefits and potential side effects) during immunization sessions was suboptimal for the most part. Our findings are discordant with that of Al-Salihi et al. in Iraq, who reported that up to 96% of primary healthcare staff informed caregivers about potential side effects during vaccination sessions [35]. This difference could be because up to 87% of immunization staff received at least one formal training course on vaccination, unlike in our study, where over two-thirds of surveyed facilities (67%) had no staff trained on immunization. A vast majority of caregivers consider health workers as their primary source of immunization information, and their recommendations are known to influence parental decisions [14, 36, 37]. Vaccination sessions offer a unique opportunity to interact with health workers and gain basic vaccine-specific information. However, immunization staff commonly need more training on interpersonal skills and their contribution to improving vaccination uptake [14].

While our study has revealed significant gaps in the knowledge, attitudes and practices of immunization staff in Cameroon, certain limitations must be considered while interpreting our results. First, the data collected from observation sessions may have been subject to some bias because EPI personnel were aware of being observed and may have performed differently from a regular unobserved day. Secondly, the knowledge indicators assessed were not comprehensive as this was done in the context of a larger assessment, narrowing the extent of the knowledge assessment. Last, but not least, given that the unit of analysis was the health facility, individual provider level variations present at facility level could have been missed. However, the nationwide coverage of our study increases the generalizability of our study findings and highlights specific regional deficits in vaccination workforce knowledge, attitudes and practices.

## Conclusion

This study highlights marked deficiencies in immunization staff training and knowledge of basic EPI indicators. It also exposes several gaps in knowledge, attitudes and practices in vaccine handling, utilization, and caregiver information sharing that could contribute to MOV, which may impact vaccination coverage and equity in Cameroon. This challenge prompts a great need to invest in systematic, comprehensive capacity building of immunization staff in Cameroon while strengthening supportive supervision and formulation of policies and strategies to minimize vaccine wastage without creating MOV.

## Data Availability

Data used for this research are available from the corresponding author upon reasonable request.

## Abbreviations

CHAI: Clinton Health Access Initiative
EPI: Expanded Program on Immunization
HCW: Healthcare workers
HF: Health facility
MCV: Measles containing vaccine
MOV: Missed Opportunities for Vaccination
WHO-MDVP: WHO Multi-dose Vaccine Policy

## References

[1] World Health Organisation. Vaccines and immunization [Internet]. 2022 [cited 2022 Dec 29]. Available from: https://www.who.int/health-topics/vaccines-and-immunization.

[2] United Nations Children’s Fund (UNICEF). Levels & Trends in Child Mortality: Report 2019, Estimates developed by the United Nations Inter-agency Group for Child Mortality Estimation (UN IGME). New York; 2019.

[3] World Health Organisation. Global Vaccine Action Plan 2011–2020. 2013.

[4] Okeibunor JC, Akanmori BD, Balcha GM, Mihigo R, Vaz RM, Nshimirimana D (2013). Enhancing access to immunization services and exploiting the benefits of recent innovations in the African region. Vaccine.

[5] Mihigo R, Okeibunor J, Anya B, Mkanda P, Zawaira F (2017). Challenges of immunization in the African Region. Pan Afr Med J.

[6] World Health Organisation. Diphtheria tetanus toxoid and pertussis (DTP3) immunization coverage among 1-year-olds, Global Health Observatory. Available from: https://www.who.int/data/gho/data/indicators/indicator-details/GHO/diphtheria-tetanus-toxoid-and-pertussis-(dtp3)-immunization-coverage-among-1-year-olds-(-).

[7] Programme Elargie de Vaccination(PEV), Ministère de la Santé, Cameroon. PEV :: Historiques. Available from: https://pevcameroon.cm/apropos/histories.

[8] LaFond A, Kanagat N, Steinglass R, Fields R, Sequeira J, Mookherji S (2015). Drivers of routine immunization coverage improvement in Africa: findings from district-level case studies. Health Policy Plan.

[9] Programme Elargie de Vaccination (PEV), Ministère de la Santé, Cameroon. Plan Pluriannuel Complet 2015–2019. 2014. Available from: https://extranet.who.int/countryplanningcycles/file-repository/CMR.

[10] National Institute of Statistics (Cameroon) and ICF. 2018 Cameroon DHS Summary Report. Maryland; 2020. Available from: www.DHSprogram.com.

[11] World Health Organization. Regional Office for Africa. Framework for the implementation of the immunization agenda 2030 in the WHO African Region: report of the Secretariat. 2021.

[12] Bobo JK, Gale JL, Thapa PB, Wassilak SG (1993). Risk factors for delayed immunization in a random sample of 1163 children from Oregon and Washington. Pediatrics.

[13] Biset G, Woday A, Mihret S, Tsihay M (2021). Full immunization coverage and associated factors among children age 12–23 months in Ethiopia: systematic review and meta-analysis of observational studies. Hum Vaccin Immunother..

[14] Shen AK, Fields R, McQuestion M (2014). The future of routine immunization in the developing world: challenges and opportunities. Glob Health Sci Pract.

[15] World Health Organisation. Quality immunization services: a planning guide. 2022.

[16] EbileAkoh W, Ateudjieu J, Nouetchognou JS, Yakum MN, DjoumaNembot F, NafackSonkeng S (2016). The expanded program on immunization service delivery in the Dschang health district, west region of Cameroon: a cross sectional survey. BMC Public Health.

[17] World Health Organisation. Global Routine Immunization Strategies and Practices (GRISP): a companion document to the Global Vaccine Action Plan (GVAP). 2016.

[18] Gavi, The Vaccine Alliance. New 2021–2025 high level strategy to leave no-one behind with immunisation approved by Gavi Board. 2022. Available from: https://www.gavi.org/news/media-room/new-2021-2025-high-level-strategy-leave-no-one-behind-immunisation-approved-gavi.

[19] Musa S, Skrijelj V, Kulo A, Habersaat KB, Smjecanin M, Primorac E (2020). Identifying barriers and drivers to vaccination: a qualitative interview study with health workers in the Federation of Bosnia and Herzegovina. Vaccine.

[20] Russo G, Miglietta A, Pezzotti P, Biguioh RM, BoutingMayaka G, Sobze MS (2015). Vaccine coverage and determinants of incomplete vaccination in children aged 12–23 months in Dschang, West Region, Cameroon: a cross-sectional survey during a polio outbreak. BMC Public Health.

[21] Ateudjieu J, Kenfack B, Nkontchou BW, Demanou M (2013). Program on immunization and cold chain monitoring: the status in eight health districts in Cameroon. BMC Res Notes.

[22] Yakum MN, Ateudjieu J, Walter EA, Watcho P (2015). Vaccine storage and cold chain monitoring in the North West region of Cameroon: a cross sectional study. BMC Res Notes.

[23] Yauba S, Sobngwi J, Jude N, Tracy B, Kobela M, Charles N (2017). Temperature monitoring in the vaccine cold chain in Cameroon. J Vacc Vacc.

[24] Yauba S, Harmelle EE, Marius VZ, Jude N, Delphine K, Calvin T (2019). Availability and status of vaccine cold chain equipment in Cameroon. J Vacc Vacc.

[25] World Bank Group. Population, total-Cameroon|Data. 2021. Available from: https://data.worldbank.org/indicator/SP.POP.TOTL?locations=CM.

[26] Presidency of the Republic of Cameroon. Cameroon. Available from: https://www.prc.cm/en/cameroon.

[27] Ministry of Public Health, Cameroon. Health Sector Strategy 2016–2027.

[28] Wallace AS, Willis F, Nwaze E, Dieng B, Sipilanyambe N, Daniels D (2017). Vaccine wastage in Nigeria: An assessment of wastage rates and related vaccinator knowledge, attitudes and practices. Vaccine.

[29] Uskun E, Uskun SB, Uysalgenc M, Yagız M (2008). Effectiveness of a training intervention on immunization to increase knowledge of primary healthcare workers and vaccination coverage rates. Public Health.

[30] Siddiqui FA, Padhani ZA, Salam RA, Aliani R, Lassi ZS, Das JK (2022). Interventions to improve immunization coverage among children and adolescents: a meta-analysis. Pediatrics.

[31] Kanagat N, Krudwig K, Wilkins KA, Kaweme S, Phiri G, Mwansa FD (2020). Health care worker preferences and perspectives on doses per container for 2 lyophilized vaccines in Senegal, Vietnam, and Zambia. Glob Health Sci Pract.

[32] World Health Organization. Reducing Missed Opportunities for Vaccination (MOV). Available from: https://www.who.int/teams/immunization-vaccines-and-biologicals/essential-programme-on-immunization/implementation/reducing-missed-opportunities-for-vaccination-(mov).

[33] Assi TM, Brown ST, Djibo A, Norman BA, Rajgopal J, Welling JS (2011). Impact of changing the measles vaccine vial size on Niger’s vaccine supply chain: a computational model. BMC Public Health.

[34] World Health Organization. WHO Policy Statement: Multi-dose Vial Policy (MDVP)- Handling of multi-dose vaccine vials after opening. Geneva; 2014. Available from: https://apps.who.int/iris/bitstream/handle/10665/135972/WHO_IVB_14.07_eng.pdf;sequence=1.

[35] Al-Salihi LG, Aakef IR, Al-Shuwaili SJ, ZakiHadi WM (2019). Primary health-care staff barriers to immunization. Indian J Community Med.

[36] Filia A, Bella A, D’Ancona F, Fabiani M, Giambi C, Rizzo C (2019). Childhood vaccinations: knowledge, attitudes and practices of paediatricians and factors associated with their confidence in addressing parental concerns, Italy, 2016. Euro Surveill.

[37] Paterson P, Meurice F, Stanberry LR, Glismann S, Rosenthal SL, Larson HJ (2016). Vaccine hesitancy and healthcare providers. Vaccine.

